# Effects of Electromagnetic Stimulation on Gene Expression of Mesenchymal Stem Cells and Repair of Bone Lesions 

**DOI:** 10.22074/cellj.2016.4870

**Published:** 2016-12-21

**Authors:** Maryam Jazayeri, Mohammad Ali Shokrgozar, Nooshin Haghighipour, Bahram Bolouri, Fereshteh Mirahmadi, Mehdi Farokhi

**Affiliations:** 1 National Cell Bank of Iran, Pasteur Institute of Iran, Tehran, Iran; 2 Department of Biophysics and Medical Physics, Iran University of Medical Sciences, Tehran, Iran

**Keywords:** Differentiation, Gene Expression, Mesenchymal Stem Cell, Osteocalcin

## Abstract

**Objective:**

Most people experience bone damage and bone disorders during their lifetimes. The use of autografts is a suitable way for injury recovery and healing. Mesenchymal stem cells (MSCs) are key players in tissue engineering and regenerative medicine. Their proliferation potential and multipotent differentiation ability enable MSCs to be considered as appropriate cells for therapy and clinical applications. Differentiation of stem cells depends on their microenvironment and biophysical stimulations. The aim of this study is to analyze the effects of an electromagnetic field on osteogenic differentiation of stem cells.

**Materials and Methods:**

In this experimental animal study, we assessed the effects of the essential parameters of a pulsatile electromagnetic field on osteogenic differentiation. The main purpose was to identify an optimum electromagnetic field for osteogenesis induction. After isolating MSCs from male Wistar rats, passage-3 (P3) cells were exposed to an electromagnetic field that had an intensity of 0.2 millitesla (mT) and frequency of 15 Hz for 10 days. Flow cytometry analysis confirmed the mesenchymal identity of the isolated cells. Pulsatile electromagnetic field-stimulated cells were examined by immunocytochemistry and real-time polymerase chain reaction (PCR).

**Results:**

Electromagnetic field stimulation alone motivated the expression of osteogenic genes. This stimulation was more effective when combined with osteogenic differentiation medium 6 hours per day for 10 days. For the *in vivo* study, an incision was made in the cranium of each animal, after which we implanted a collagen scaffold seeded with stimulated cells into the animals. Histological analysis revealed bone formation after 10 weeks of implantation.

**Conclusion:**

We have shown that the combined use of chemical factors and an electromagnetic field was more effective for inducing osteogenesis. These elements have synergistic effects and are beneficial for bone tissue engineering applications.

## Introduction

Stem cells stay in their undifferentiated stage until they receive appropriate activation signals and begin the differentiation process into specific lineages, according to the type of received stimuli. 

Signals such as growth factors and physical cues are provided by the surrounding cellular microenvironment (1,6). Cell density and cell-cell interactions play major roles in the differentiation process (7). Some chemical compounds such as dexamethasone are osteogenic supplements and, like growth factors, they play essential roles in osteogenic differentiation of mesenchymal stem cells (MSCs) (8). In this study, we have used a pulsed electromagnetic field (PEMF) as the biophysical guide to create waves with constant properties. Such waves are non-ionizing and create non-thermal fields with high rates of amplitude changes (9). The applied frequency of the extremely low frequency electromagnetic field is under 300 Hz and the amplitude ranges from 0.2 to 20 millitesla (mT) (10). PEMF is clinically used to treat osteoporosis by increasing bone mass in women with menopause and snapback in patients with osteotomies. This field also acts to reduce the bone resorption activity of osteoclasts (11) as well as increase calcium content and other bone minerals (12). As a biophysical factor, PEMF motivates the release of Ca^2+^ions from the smooth endoplasmic reticulum as the starting point of signaling pathways that activate osteogenic differentiation. The increase in intracellular Ca^2+^level also triggers enzymatic cascades, resulting in the secretion of growth factors such as bone morphogenetic proteins (BMPs), expression of osteoblast-specific genes, and cell proliferation (13). The interaction between an electromagnetic field and biological tissue is related to the amplitude, frequency, and form of the wave in addition to the time duration of the exposure (9).

Until now, modern medicine has used extremely low frequency PEMFs to treat non-union bone fractures, pseudarthrosis, osteoporosis, and periodontal disease (9). Interaction of electromagnetic fields with the extracellular matrix can increase cytosolic Ca^2+^and then promote the proliferation of osteoblastic cells (14). It has been proven that the expression of osteoblastic marker genes is upregulated in response to a combination of specific PEMFs and chemical compounds such as BMPs or other inductive factors (9, 15).

In this study, we researched the effect of PEMF on MSCs proliferation and differentiation toward osteoblasts along with the amount of expression of osteoblastic marker genes such as osteocalcin (*Ocn*) and runt-related transcription factor 2 (*Runx2*). Our objective was to analyze the effects of an electromagnetic field on osteogenic differentiation of stem cells. In addition, we assessed the influence of chemical factors when combined with PEMF. 

## Materials and Methods

This was an experimental animal study conducted on rat bone marrow derived MSCs.

### Mesenchymal stem cell isolation and culture

All animal experiments were performed according to approved guidelines of the Ethics Committee at Pasteur Institute of Iran. A total of 9 male, 4-week-old Wistar rats (weights: 230-250 g) were anesthetized in order to obtain bone marrow aspiration from their iliac crests under sterile conditions. After isolation of bone marrow stem cells according to the Ficoll-Paque technique, we cultured these cells in Minimum Essential Medium Eagle Alpha Modification (α-MEM medium, Sigma, NY, USA) supplemented with 15% fetal bovine serum (FBS), 1% penicillin/ streptomycin [100 U/ml of penicillin and 100 µg/ml of streptomycin (Sigma, NY, USA)] and 1% L-glutamine (Gibco, NY, USA). The medium was changed every 3 days (8). Cells at passage-3 (P3) were used for the experiments. The rat osteosarcoma cell line (UMR106) provided by National Cell Bank of Iran (C586) was the positive control group and stem cells comprised the negative control group.

### Multipotential assay

We performed chondrogenic, osteogenic, and adipogenic differentiation experiments to examine the multipotential differentiation ability of the isolated cells. For osteogenic differentiation P3 cells were exposed to osteogenic medium that contained Dulbecco’s Modified Eagle’s Medium (DMEM), 10% FBS, 100 nM dexamethasone, 10 mM β-glycerol phosphate, and L-ascorbic acid 2-phosphate for 21 days. The medium was changed every 3 days. Thereafter, the cells were fixed and stained with Alizarin red S.

In order to induce adipogenesis, the cells were subjected to DMEM that contained 10% FBS, 0.5 μM of 3-isobutyl-1-methylxanthine (IBMX), 1 μM dexamethasone, 10 μg/ml insulin, and 100 μM indomethacin for 15 days. Subsequently, the cells were fixed with 4% paraformaldehyde and stained with Oil red O.

For directing cells toward chondrogenic differentiation, the cell pellets were prepared and incubated with DMEM that contained 50 mM ascorbic acid-2 phosphate, 10 ng/mL transforming growth factor b1 (TGF b1, R&D Systems, USA), 100 nM dexamethasone, 1% ITS-Premix (BD Biosciences, USA), and 1 mM sodium pyruvate (Gibco, NY, USA) for 28 days. Chondrogenic differentiation was examined by fixing the cells with 10% formalin, followed by sectioning the pellets and staining them with Alcian blue. All chemicals unless otherwise indicated were purchased from Sigma, USA (16).

### Immunophenotyping

Bone marrow derived MSCs were studied for the expression of CD45 as the hematopoietic marker, along with CD73 and CD90 as the MSC surface markers. CD73, a PE conjugated antibody, (BD Biosciences, CA, USA) and FITC-conjugated goat anti-mouse IgG antibodies for CD45 and CD90 (FITC conjugated) were used. Mouse IgG1 K isotype control (eBiosciences, CA, USA), mouse IgG2a K isotype control FITC (eBiosciences, CA, USA), and donkey anti-mouse IgG (H+L) PE (eBiosciences, CA, USA) were used as secondary antibodies for detection of the selected markers. Unstained cells were used for gating in the flow cytometric analysis. We counted 15000 events for each antibody. Data were analyzed by FlowJo software version 7.6.4 (17).

### Pulsed electromagnetic field exposure

PEMF stimulation was performed using Helmholtz coils of copper wire (18). A pair of 12.7 cm-diameter circular coils was placed opposite to each other within the incubator and the cell flask located in the uniform field area of the coil center. The proper shielding and utilization of Plexiglass was performed to guarantee the prevention of any disturbance to the applied stimulating magnetic field. The electromagnetic field generator, named the Helmholtz coil, consists of two solenoid electromagnets on the same axis. The non-sinusoidal magnetic field is generated by an electric current through the coils. This device is used to produce uniform electromagnetic waves in order to create a uniform magnetic field. These coils cancel the interference of external magnetic fields generated by nearby electrical devices or the Earth’s magnetic field.

The employed device had three parts: a stimulator, the coils, and a control box. Intensity of the created field was regulated by changing the voltage of the stimulator. The apparatus was ordered by the National Cell Bank of Iran and Behi Afzar Saz Pooya Company of Iran fabricated the entire system (10).

Intensity of the field was 0.2 mT with a 15 Hz frequency. We used a pulse on time of 40 mseconds and pulse off time of 27 mseconds. A tesla meter (Lutron) measured the magnetic flux at the center of the coil. At first, we analyzed three differentiation periods of daily exposure in order to determine the most effective duration of exposure. PEMF was used to stimulate the cells with 0.2 mT and 15 Hz for 10 consecutive days with 2, 4, and 6 hours of exposure per day. The cells from all the groups were exposed to PEMF at 0.2 mT intensity and 15 Hz frequency for 10 consecutive days and 6 hours of exposure per day. This study had three experimental groups: i. Cells incubated with regular culture medium and exposed to the field, ii. Cells stimulated with simultaneous application of the electromagnetic field and chemical differentiation medium (50 μM ascorbate-2 phosphate, 10 mM β-glycerophosphate, and 0.1 μM dexamethasone) for 7 consecutive days, and iii. Cells subjected to a combination of the mentioned electromagnetic field and chemical differentiation medium for 10 consecutive days.

Upon completion of the tests, we performed real- time polymerase chian reaction (PCR) analysis to quantify the expressions of the marker genes (15, 19). Untreated MSCs were utilized as the negative control and UMR-106 was the positive control.

### MTT assay 

The tetrazole 3-(4,5-dimethylthiazol-2-yl)- 2,5-diphenyltetrazolium bromide (MTT), as a histomorphological stain, was used to study the effect of PEMF on MSC proliferation. MTT is reduced to purple formazan by viable cells. Hence, the number of living cells can be determined based on the absorbance of the formazan solution (20). We have performed the MTT assay on cells subjected to the 0.2 mT electromagnetic field (6 hours of exposure per day) on the 5^th^, 10^th^, and 14^th^days.

### Immunocytochemistry

Immunocytochemistry assay was used to scan the influence of electromagnetic field exposure. Antibodies were used against two osteogenic markers, anti *Runx2* and anti osteocalcins. Immediately after exposure to the field, the cells were washed twice with phosphate-buffered saline (PBS) and fixed with 4% paraformaldehyde (Sigma, NY, USA) for 20 minutes at 4˚C. Next, they were permeabilized with 0.5% Triton X100 (Merck, NJ, USA) after which 0.5% gout serum was used to block the nonspecific antibodies. Cells were incubated overnight at 4˚C with mouse monoclonal antibodies against *Runx2* and *Ocn* (both from Abcam, Cambridge, UK). Thereafter, they were incubated with FITC conjugated secondary antibody at a 1:100 dilution (Abcam, Cambridge, UK) at room temperature in the dark for 2 hours. Finally, the presence of the mentioned proteins was examined under a Zeiss fluorescence microscope (×630) (21).

### Real-time reverse transcriptional polymerase chain reaction assay 

We used real-time reverse transcriptional polymerase chain reaction (RT-PCR) to examine the expressions of the *Ocn* and *Runx2* genes by the stimulated cells. Total RNA was extracted using the RNeasy plus Mini Kit (Qiagen, MD, USA) according to the manufacturer’s instructions. The purity of extracted RNA was evaluated by means of a nanodrop spectrophotometer (Implen, Germany). High quality samples with concentrations >400 ng/μl and A260/A280 ≥1.8 were chosen for analysis. The QuantiTect Reverse Transcription Kit (Qiagen, MD, USA) was used to synthesize complementary DNA (cDNA) from the extracted RNA. Gel electrophoresis was carried out to verify the integrity of cDNA. TaqMan real-time PCR was performed for quantitative analysis of *Ocn* and *Runx2* expressions. Reactions were carried out using an ABI StepOne system with StepOne v2.1 software (Applied Biosystems, CA, USA).

All primers and probes were designed using the Primer Express software (version 3.0). The recommended sequences by this software were analyzed using gene runner software. Ribosomal protein large subunit 13a (*RPL13A*) was selected as the housekeeping gene for normalization of the obtained data that corresponded to *Runx2* and *Ocn* mRNA level quantification. Primer sequences were as follows: 

*Runx2*

 F: 5ʹ-GCCAGGTTCAACGATCTGAGA-3ʹ

 R: 5ʹ-GGAGGATTTGTGAAGACCGTTATG-3ʹ 

probe:

5ʹ-TGAAACTCTTGCCTCGTCCGCTCC-3ʹ

*Ocn*

 F: 5ʹ-GCAGACCTAGCAGACACCATGA-3ʹ

 R: 5ʹ-CCAGGTCAGAGAGGCAGAATG-3ʹ

 probe:

 5ʹ-TCTCTGCTCACTCTGCTGGCCCTG-3ʹ

*RPL13*

 F: 5ʹ-TGAACACCAACCCGTCTCG-3ʹ

 R: 5ʹ-GCAGCCTGGCCTCTTTTG-3ʹ 

 probe:

 5ʹ-CCCCTACCACTTCCGAGCCCCA-3ʹ.

PCR products were checked by gel electrophoresis according to the product size (data not shown). Each reaction was performed in triplicate with a total volume of 20 μl that contained 5 μl of cDNA sample, 10 µl of TaqMan Universal PCR Master Mix (Applied Biosystems, USA), 10 pmol of each primer, and 5ʹ-Fam-/3ʹ-Tamra-labeled probe. The thermal cycling profile involved an initial activation for 10 minutes at 95˚C, followed by 15 seconds at 95˚C, 1 minute at 60˚C, and running for 40 cycles. The melting curve stage was set at 95˚C (15 seconds), 60˚C (1 minute), and 95˚C (15 seconds) (22). Gene expression values were calculated using the following formula:

ΔΔCT= [minimum CT Targets-minimum CT RPL-13A]Test samples-[minimum CT Targets- minimum CT RPL-13A]Stem cells

Real-time PCR was performed to compare the effects of 0.1 and 0.2 mT-fields on the expressions of the osteogenic markers. In another part of this study the effects of three daily-exposure durations of 2, 4 and 6 hours for electromagnetic field application were studied after 10 days of stimulation. Real-time PCR was used to compare gene expression levels among the above mentioned groups.

### Surgical procedures 

Each animal was anesthetized and a small incision was made on the left side of the cranium. The periosteum and soft tissues were removed to access the cranial bone. The collagen-based scaffolds (23) with dimensions of 5×5×1 mm^3^were implanted after a predrilling with a dental drill. The following three groups were defined and studied in triplicate: i. Bone sockets without any scaffolds; ii. Defects filled with scaffolds seeded with untreated MSCs, and iii. Defects filled with scaffolds seeded with electromagnetically and chemically motivated MSCs. Vicryl 3-0 suture was used to close the incisions. We performed autologous transplantation and each animal used its own stem cells. The scaffolds were retrieved after 10 weeks of implantation and fixed with 4% paraformaldehyde for 12 hours. Thereafter, decalcification of bones was carried out in 10% EDTA for two weeks followed by embedding in paraffin. Tissue blocks were sectioned into 5 µm thick sections and stained with hematoxylin and eosin (H&E) to assess bone healing (24).

### Statistical analysis 

All data that corresponded to the three separate experiments were expressed as means ± SD. Statistical analyses were performed using oneway ANOVA and the student’s t test via SPSS software version 17.0. P values lower than 0.05 were considered statistically significant.

## Results

### Differentiation potential assays

The results of multi-lineage differentiation experiments confirmed the potential of isolated cells to differentiate into adipocytes, osteoblasts, and chondrocytes. After oil red O staining, we observed the presence of lipid vacuoles. Alizarin red S staining revealed the presence of calcified nodules and Alcian blue staining demonstrated sulfated glycosaminoglycans and chondrogenesis (data not shown). These observations indicated the multi-potent identity of the isolated cells.

### Characterization of mesenchymal stem cells

**Fig.1:**
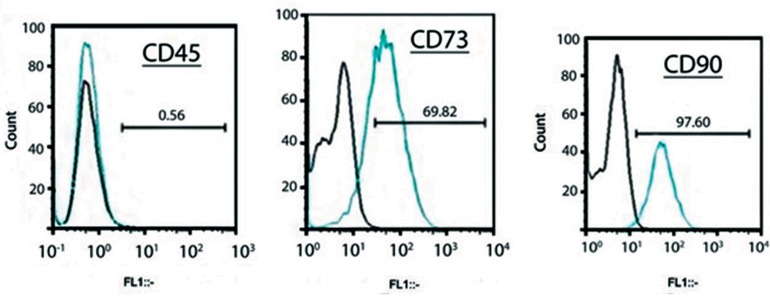
Flow cytometric analysis of isolated rat bone marrow-derived mesenchymal stem cells (MSCs). Cells were analyzed for expression of MSC specific surface markers. A. CD45 (negative marker), B. CD73 (positive marker), and C. CD90 (positive marker) as well as cell size (forward-angle light scatter, FAS). The positive mean value of each marker is shown in the corresponding graph. Graphs confirm the mesenchymal identity of the isolated cells.

We used flow cytometry to characterize the mesenchymal identity of the isolated cells. According to the results, the cells were negative for the hematopoietic marker, CD45. These cells highly expressed CD73 and CD90 as MSC- associated surface proteins. The obtained results (Fig.1) confirmed the mesenchymal identity of the isolated cells. 

### Electromagnetic field and proliferation

**Fig.2:**
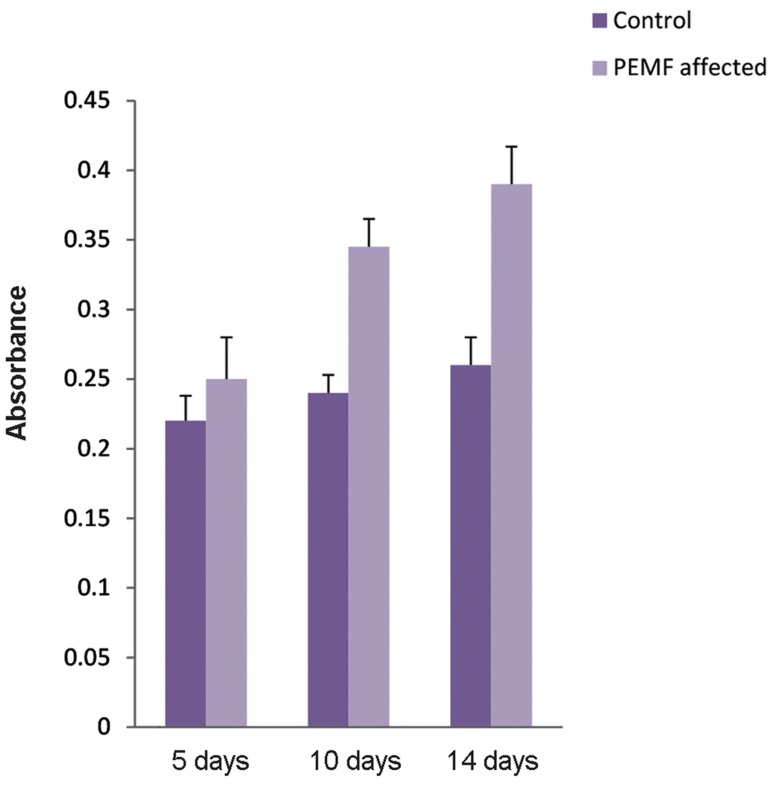
Results of the MTT assay on mesenchymal stem cells (MSCs) exposed to an electromagnetic field (0.2 mT, 15 Hz, 6 hours/day) to estimate the number of cells after 5, 10 and 14 days. Unstimulated cells cultured for 5, 10, and 14 days were used as the control groups (P<0.05). PEMF; Pulsed electromagnetic field.

We used the MTT assay to determine the influence of a low frequency electromagnetic field on stem cell proliferation after 5, 10, and 14 days of cell exposure to PEMF (0.2 mT, 15 Hz for 6 hours exposure/day). Unstimulated MSCs were the negative control. As illustrated in Figure 2, cells stimulated with the electromagnetic field had a higher proliferation rate compared to unstimulated MSCs. Thus PEMF treatment for 14 days did not have any negative effect on MSC proliferation; rather, it enhanced the proliferative activity of these cells.

### Effects of electromagnetic field intensity 

**Fig.3:**
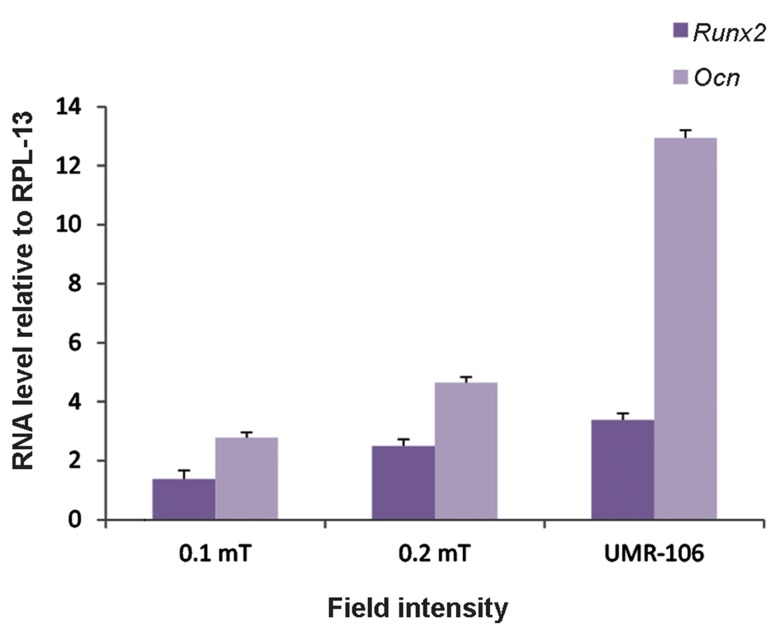
The effects of two different electromagnetic field intensity levels (0.1 mT and 0.2 mT) at 15 Hz, 6 hours/day for 10 consecutive days on the expressions of *Runx2* and *Ocn* according to realtime polymerase chain reaction (PCR). UMR-106 was the positive control. As shown, 0.2 mT intensity was more influential in stimulating mesenchymal stem cells (MSCs) to express osteogenic markers (P<0.05).

We conducted real-time PCR analysis of the effects of field intensity on gene expression. MSCs from two separate groups were exposed to 0.1 mTor 0.2 mT-intensity fields with similar field parameters of 15 Hz frequency and 6 hours application of PEMF per day for 10 consecutive days. As shown in Figure 3, the 0.2 mT intensity field resulted in a greater increase in expression of osteoblastic genes compared to the 0.1 mT field.

### Effects of electromagnetic field exposure duration

**Fig.4:**
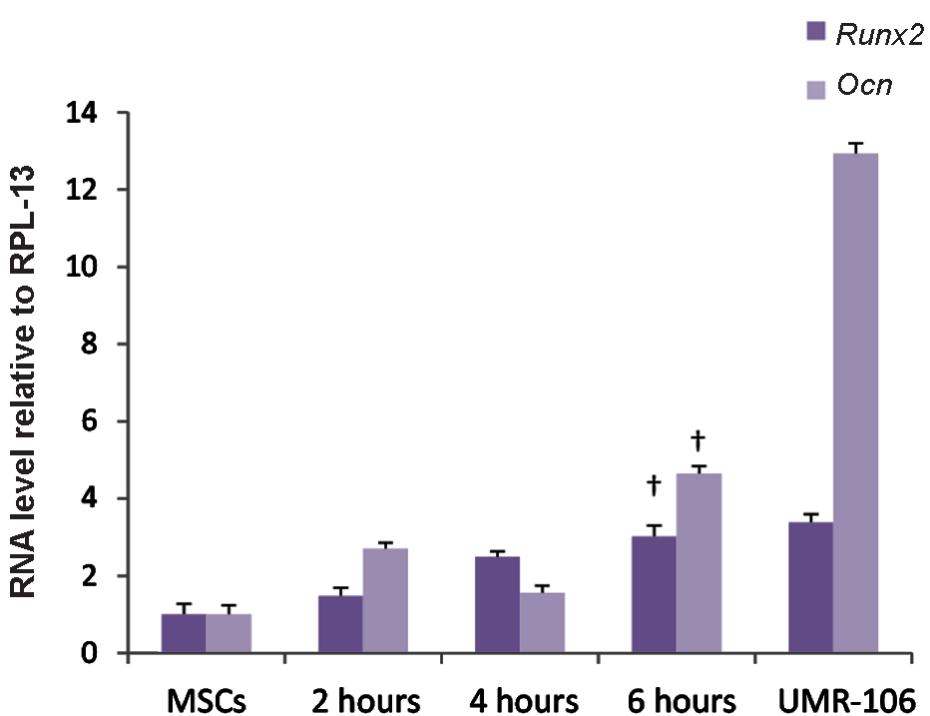
The effect of exposure duration (2, 4 or 6 hours/day) of the electromagnetic field (0.2 mT, 15 Hz, for 10 days) on osteoblastic gene expressions. UMR-106 and untreated mesenchymal stem cells (MSCs) were the positive and negative controls, respectively. The 6 hours of exposure per day was the most effective time duration (P<0.05 in † and P<0.001 in other columns).

We tested three different durations of daily exposure in order to find the most influential duration. Stem cells were stimulated with PEMF (0.2 mT and 15 Hz) for 10 consecutive days with daily exposure durations of 2, 4, or 6 hours. We observed the highest expression levels of *Runx2* and *Ocn* in the group that received 6 hours of daily exposure to PEMF (Fig.4). 

### Combination of electromagnetic field and chemical induction

**Fig.5:**
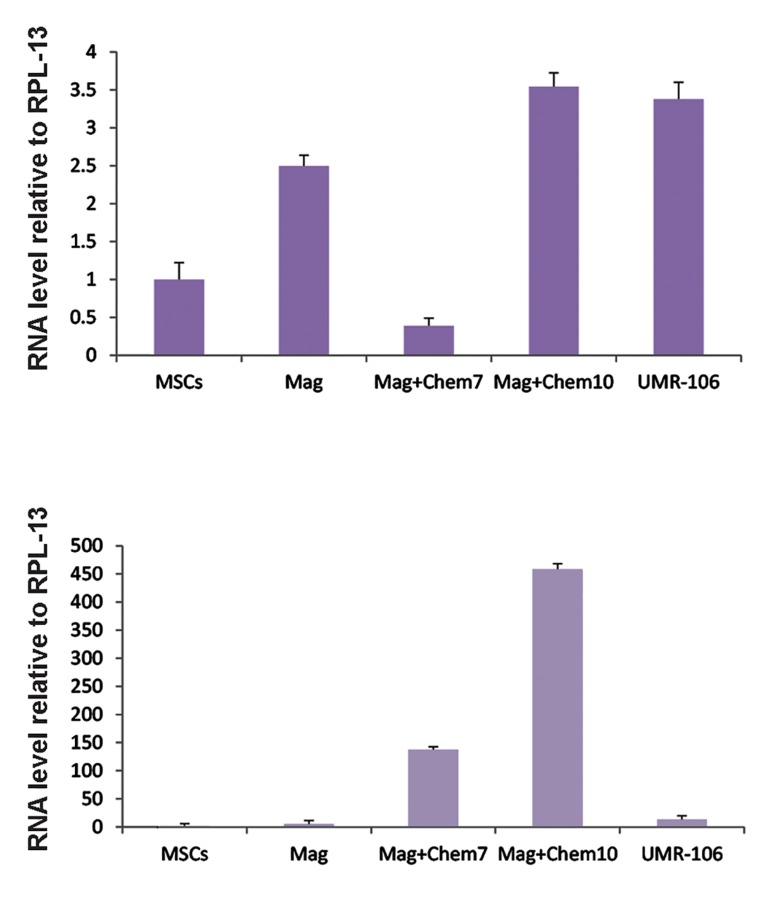
Osteoblastic gene expression levels by cells simultaneously subjected to electromagnetic field and induction medium for 7 or 10 days, or only exposed to the electromagnetic field (magnetic group) for 10 days. An electromagnetic field (0.2 mT, 15 Hz) was applied for 6 hours per day. UMR-106 and untreated mesenchymal stem cells (MSCs) were used as positive and negative controls, respectively. A. *Runx2* and B. *Ocn*. Combined application of induction medium and pulsed electromagnetic field (PEMF) for 10 days was the most effective treatment (P<0.001). Mag; Electromagnetic stimulation and Chem; Chemical induction.

Simultaneous application of chemical supplements and the electromagnetic field was carried out to assess the effects of combined treatment on expressions of the osteogenic genes. Real-time PCR was performed after electromagnetic field exposure at 6 hours daily for a 10-day period along with concurrent incubation with chemical factors in order to quantify mRNA levels of the osteogenic markers. MSCs were incubated for 7 and 10 days in induction medium. We compared the results with cells stimulated only with PEMF. The results showed that *Runx2* and *Ocn* had the highest expression levels 10 days after cells were subjected to the combination of induction medium and PEMF waves (Fig.5A, B). 

### Immunocytochemistry for pulsed electromagnetic field stimulation 

**Fig.6:**
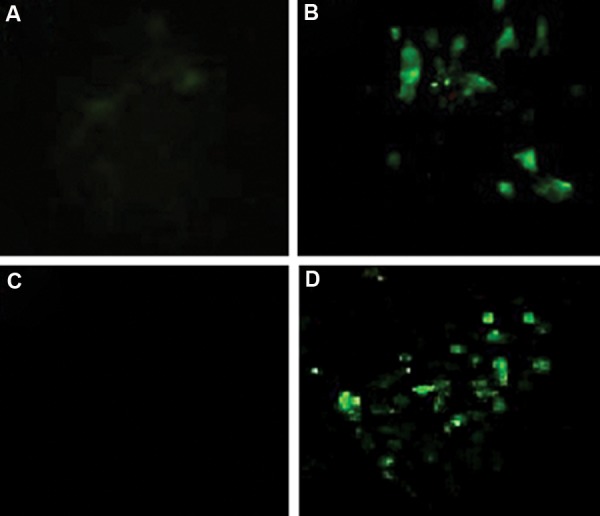
Immunocytochemistry to localize. A. *Runx2* in unstimulated stem cells, B. *Runx2* in cells exposed to electromagnetic field, C. Osteocalcin in unstimulated mesenchymal stem cells (MSCs), and D. Osteocalcin in cells only exposed to the electromagnetic field (0.2 mT, 15 Hz, 6 hours/day for 10 consecutive days). Electromagnetic field is solely able to promote the expression of osteogenic genes and osteogenic differentiation. Fluorescence visualization was performed using a Carl Zeiss fluorescent microscope (×630).

Immunocytochemistry results demonstrated a slight expression of *Runx2* protein in stem cells (Fig.6A) and presence of higher amounts of *Runx2* in cells stimulated only with the electromagnetic field (Fig.6B). We observed no osteocalcin expression in unstimulated stem cells (Fig.6C) and large amounts of osteocalcin in cells stimulated only with the electromagnetic field (Fig.6D). 

### In vivo studies

**Fig.7:**
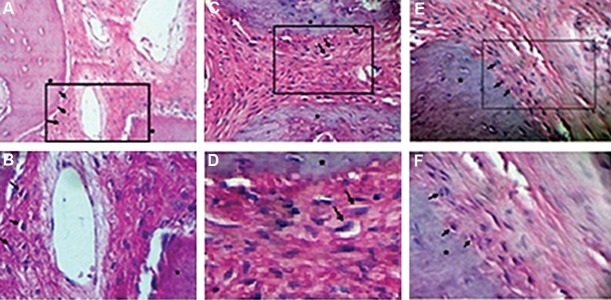
Histological analysis of *in vivo* bone formation using hematoxylin and eosin (H&E) staining. A, B. Bone sockets in the absence of scaffolds as the negative control, C, D. Defects filled by undifferentiated mesenchymal stem cell (MSC)-seeded scaffolds, E, and F. Defects filled by electromagnetically differentiated MSC-seeded scaffolds. Arrows show osteoblast cells. Newly formed bones (rectangular area) are located adjacent to the pre-existing bones (*).

Histological analysis was performed to assess bone and tissue ingrowth by differentiated MSCs stimulated by the electromagnetic field. After 10 weeks of implantation, we observed no signs of any inflammatory cells such as macrophages, lymphocytes, or giant cells in the different experimental groups. In all test groups, new osteoid areas formed adjacent to the pre-existing bones (Fig.7). As a result of osteoblast activity, osteoids were produced on the surface of the new bone. Implanted scaffolds underwent degradation and no signs of the scaffold residues could be observed. In general, after 10 weeks the created defects had evidence of new bone in all three test groups.

## Discussion

The present study evaluated the effects of electromagnetic field application and biochemical stimulation on MSCs and their gene expression patterns. Electromagnetic field parameters were selected such that the effect of PEMF on the expression of osteogenic markers and osteogenic differentiation could be assessed. We used the MTT assay, immunocytochemistry, TaqMan real- time PCR, and histological analysis to study the behavior of stem cells in response to this exposure.

Flow cytometry analysis confirmed the identity of the cells .The MTT assay was carried out to investigate the effects of the low electromagnetic field on MSCs. The results indicated a progressive increase in the proliferation rate of MSCs due to the application of the extremely low frequency PEMF. A 20-60% increase in cell density due to exposure to the field was previously reported (15, 19). It has been suggested that the healing effects of the electromagnetic field on fractures may be related to its effects on promoting proliferation and growth acceleration in stem cells and preparing more progenitor cells for differentiation toward osteoblasts (19).

Previous studies have suggested that PEMF may activate free ions on the cell surface. K^+^and Ca^2+^currents affect the activated K^+^channels when progressing from the G_1_-to S-phase and this mechanism may promote the proliferation of undifferentiated stem cells (14). In another study, electromagnetic fields have been shown to alter membrane functions by opening or closing ion channels, bind ligands, and the numbers and distribution of receptors (11). Thus PEMFs affect the molecular currents and cause a specific transmembrane signaling which can promote osteogenic differentiation.

There are contradictions in terms of the duration of daily exposure and its consequences among different studies. Although Matsumoto et al. (25) have reported that longer stimulation durations per day resulted in higher bone contents, they did not observe any significant difference in terms of bone formation between two groups stimulated for 4 or 8 hours per day. However, the results of the present experiment revealed that 6 hours of stimulation per day showed greater benefit in enhancing the mRNA level of *Ocn*, as a late osteogenesis marker.

Previous studies have demonstrated that compared to higher intensity values such as 0.8 mT, low electromagnetic intensities (0.2 and 0.3 mT) are more effective in promoting bone formation (25). In the present study, we have compared low electromagnetic intensities in order to determine which one more effectively promoted osteogenesis. Among the 0.1 and 0.2 mT intensities, we determined that the latter intensity level led to higher expression levels of early and late osteoblastic genes. These findings supported the results of previous reports (26, 27).

*Runx2* has an inconsistent expression pattern during differentiation. This disharmony in mRNA levels was first reported by Tsai et al. (15). However, an overall up-regulation of this gene during osteogenic culture has been observed. Jansen et al. (28) previously reported that bone marker genes reached their highest expression levels between days 5 and 9 of exposure to PEMF. In other words, these genes reached their maximum expression levels just before and around the onset of cell mineralization. This result was also observed in the current study, in which we documented the highest expressions of *Runx2* and *Ocn* on day 10 of exposure to the electromagnetic field. *Runx2* and *Ocn* expressions downregulated between days 10 and 14 which indicated a transition to the mineralization stage. According to this finding and in agreement with previous reports, we concluded that PEMF treatment affected osteogenic differentiation of stem cells and stimulated mineralization at a time period just prior to the mineralization stage. Downregulation of osteogenic genes after an initial upregulation has been reported in previous works (15, 28).

Multiple signaling pathways promote osteogenic differentiation of stem cells, some of which such as the canonical Wnt signaling pathway are triggered by PEMF application. The canonical Wnt pathway results in β-catenin stability, which goes to the nucleus and leads to the expression of target genes subsequently resulting in osteogenic differentiation and bone formation (20, 29). The chemical induction medium that contained ascorbic acid, β-glycerophosphate, and dexamethasone has promoted mineralization of the extracellular matrix through activation of different signal transduction pathways (8). Thus PEMF waves and the utilized biochemical factors reinforced the effects of each other.

Implantation of differentiated cells on prefabricated scaffolds to defective areas of the bone and following the changes in the tissue has not been previously considered. According to the *in vivo* results of this study, differentiated osteoblasts seeded on scaffolds promoted filling of the incision and healing of the defects, after H&E staining of the sections related to different implant types, we observed the formation of new bone tissues throughout the scaffold structures. There was no fibrous tissue formation or inflammatory response observed in the different groups. The new osteoblasts produced osteoids on the surface of the pre-existing bone.

This research intended to find the optimized parameters of the electromagnetic field in order to achieve an osseous tissue that could be implanted into the stem cell donor. In this process certain defects or malformations would be treated, therefore PEMF could be used to treat some osteogenic disorders via promoting osteogenic differentiation. In similar studies, no *in vivo* analysis was used to estimate the efficiency of the new osteoblasts and their life-time. Some of the field parameters utilized in those studies were slightly different. 

## Conclusion

The induced electric currents by electromagnetic fields have the potential to induce osteogenesis in MSCs. Therefore, PEMF has modulating effects on stem cell proliferation and promotion of osteogenic differentiation. PEMF is a potentially low cost tool for tissue engineering which can construct new bone. This tool can be applied for fabrication of autografts in orthopedic surgeries as well as for treatment of maxillofacial disorders. 
